# Psychometric structure and score interpretation of the SCL-90 among Chinese first-year undergraduates: an analysis of an open dataset

**DOI:** 10.3389/fpsyg.2026.1913286

**Published:** 2026-09-30

**Authors:** Jinglei Wen, Yujiao Deng

**Affiliations:** 1 Law School, Hengyang Normal University, Hengyang, Hunan, China; 2 School of Intelligent Manufacturing, Wuhan Railway Vocational College of Technology, Wuhan, Hubei, China

**Keywords:** bifactor model, general severity factor, internal replication, open data, ordinal confirmatory factor analysis, psychometric structure, SCL-90, score interpretation

## Abstract

**Introduction:**

The Symptom Checklist-90 (SCL-90) provides total and domain scores whose shared and distinct information remains debated.

**Methods:**

We analyzed an open dataset of 14,371 Chinese first-year undergraduates in calibration and validation halves. Four item-level structures (unidimensional, nine correlated factors, second-order, and orthogonal bifactor) were compared on the same 83 domain-scored items using robust maximum likelihood (MLR) in both halves and mean- and variance-adjusted weighted least squares (WLSMV) for ordered items in validation. A separate single-factor model included all 90 items. Evaluation combined absolute and relative fit, numerical diagnostics, loadings, residuals, and general and specific reliability.

**Results:**

For the 83-item comparison, the correlated-factor model had a non-positive-definite latent matrix. The bifactor model improved on the admissible second-order model, but its WLSMV comparative fit index (CFI) advantage was only 0.0079; MLR validation fit remained mixed (CFI = 0.849, Tucker-Lewis index [TLI] = 0.842, root mean square error of approximation [RMSEA] = 0.045, standardized root mean square residual [SRMR] = 0.042). Under that MLR bifactor specification, general explained common variance (ECV) was 0.813 [0.804, 0.822] and omegaH was 0.963 [0.961, 0.965]. Specific omegaHS ranged from 0.017 to 0.293, with somatization, phobic anxiety, and hostility retaining comparatively more specific information. WLSMV general omegaH was 0.967 and specific omegaHS ranged from 0.005 to 0.186. The 90-item single-factor model showed limited incremental fit (MLR CFI = 0.769/0.773; WLSMV CFI = 0.896); RMSEA was 0.052/0.052 and 0.044, and residual dependence persisted. High 90-item alpha (0.978) therefore coexisted with departures from strict unidimensionality. An exploratory mixture audit yielded no stable class solution.

**Discussion:**

These data support a substantial general-severity component and uneven domain-specific information within the modeled 83-item structure. Its reliability decomposition does not establish that of the conventional 90-item total or global severity index. Independent-sample replication remains necessary.

## 1 Introduction

The Symptom Checklist-90 (SCL-90) is a 90-item self-report inventory of symptom distress, originally developed for outpatient assessment (Derogatis et al., 1973). Its nine domains cover somatization, obsessive-compulsive symptoms, interpersonal sensitivity, depression, anxiety, hostility, phobic anxiety, paranoid ideation, and psychoticism; seven additional items contribute to overall scoring without belonging to these domains. Responses use five ordered categories of increasing distress. Chinese implementations commonly use 1–5 scoring, and the instrument remains widely used in university-student research (Dang et al., 2021; Yu et al., 2019, 2020). Within this familiar scoring scheme, the distinct information conveyed by domain scores and the implications of their shared variance for a global score remain unresolved. Questions about Chinese item wording and norms further shape this interpretation (Dang et al., 2021; Zhou et al., 2021).

Four model families distinguish different sources of symptom covariance. A unidimensional model attributes item covariance to one common factor. A correlated nine-factor model retains the conventional domains and allows their associations to vary. A second-order model explains those associations through a higher-order factor, whereas an orthogonal bifactor model represents general and domain-specific contributions directly at the item level. Each is a statistical representation of symptom covariance; none, by itself, demonstrates either nine distinct disorders or one underlying psychological entity.

Direct SCL-90 studies have yielded findings that depend on specification, analytic method, and sample. Vassend and Skrondal (1999) found inadequate overall fit for pre-specified item-level structures and showed that dimensionality judgments depended on the analytic method, criteria, and level of negative affectivity. Urbán et al. (2014, 2016) obtained their most favorable fits with a bifactor extension allowing specific factors to correlate. General and specific information varied across their community and clinical samples. Those findings concern an extended bifactor representation whose correlated specifics differ from a fully orthogonal specification.

Chinese evidence includes direct comparisons of these model families. In a police sample, Chen et al. (2020) reported a small advantage for the nine-specific-factor bifactor over the correlated nine-factor model (comparative fit index [CFI] 0.980 versus 0.979; root mean square error of approximation [RMSEA] 0.077 versus 0.079); the unidimensional model had CFI 0.975 but RMSEA 0.093, and the second-order models did not converge. The fit ranking thus coexisted with differences between indices. In Cypriot adolescents, Gomez et al. (2026) found that the classic bifactor confirmatory factor analysis (CFA) did not converge and preferred exploratory structural equation modeling (ESEM), which allows cross-loadings, over conventional CFA representations. Model constraints and admissibility therefore matter alongside the fit indices when assessing validation evidence for the scale.

Student studies identify a further distinction between internal consistency and domain-specific information. Yu et al. (2019) reported poor discriminant validity among Chinese undergraduates despite high reliability. Their published fit diagram uses observed domain scores and residual correlations, limiting direct comparison with the item-level models examined here. In French first-year students, Lignier et al. (2024) found substantial general-distress information and uneven specific reliability, with somatization, hostility, and phobic anxiety among the more robust domains. Their favorable ESEM solutions allowed cross-loadings; the reported bifactor CFA and ESEM loading tables also differed in coverage of the seven additional items. Applying these findings to a particular score therefore requires attention to the item set and model constraints as well as fit.

Bifactor flexibility can improve relative fit without ensuring interpretable or sufficiently reliable specific factors (Bonifay et al., 2017; Bornovalova et al., 2020; Watts et al., 2024). Score interpretation therefore requires attention to absolute fit, admissibility, general and specific loadings, residual structure, and reliability estimates with score-appropriate denominators (Reise, 2012; Reise et al., 2013; Rodriguez et al., 2016). Person-centered models pose a related interpretive problem: covariance restrictions, non-normality, or unstable enumeration can produce partitions that need not correspond to distinct symptom subtypes (Bauer and Curran, 2003; Choi and Hong, 2022; Lubke and Neale, 2006; Masyn, 2013; Nylund et al., 2007). We accordingly treat mixture analysis as an exploratory audit of model dependence, with clinical classification outside its scope.

Using an openly licensed dataset of 14,371 Chinese first-year undergraduates, this structured secondary analysis compares the four model families on the same 83 domain-scored items, integrating absolute and relative fit, targeted admissibility checks, general and specific reliability, and residual structure. Random halves and continuous-item versus ordinal estimation assess internal stability. A separate 90-item single-factor check evaluates a strict representation of the complete inventory; the 83-item reliability decomposition remains specific to that modeled score rather than the conventional total or Global Severity Index (GSI). Existing descriptive thresholds and the mixture audit are secondary. Building on established model families and general-distress interpretations, the study adds a score-specific assessment of these data with explicit distinctions between the item sets and their modeled score properties.

## 2 Methods

### 2.1 Design and data source

This structured, cross-sectional secondary analysis used the publicly available Mendeley Data deposit by Wang (2025); (DOI 10.17632/btzgmr2rt2.1; CC BY 4.0), without new recruitment, data collection, or individual-level linkage. The deposit describes the participants as Chinese first-year undergraduates from two provinces. Its association with Wang et al. (2026) was checked against the public OSF file linked by that publication: all 113 shared fields agreed record-for-record across 14,372 raw records. The files were not byte-identical because the OSF file contained additional fields; those fields were not imported, and the Mendeley file remained the analysis input.

According to the associated publication, data were collected electronically in October–November 2023 alongside routine mental health assessments of incoming students (Wang et al., 2026). The source authors reported coverage of all incoming students at one institution; at the other, approximately half of the departments were randomly selected and all eligible first-year students within those departments were invited. The available documentation did not establish how the institutions were selected, invitation denominators or response rates, or whether participation was voluntary or mandatory. Recruitment procedures could not be mapped securely to the numeric location codes. We retain the sources’ undergraduate designation as a source description; the educational classification and actual administration procedures were not independently verified.

The study was not preregistered: its analysis protocol was documented during manuscript development rather than publicly fixed before fitting. The ordinal four-model comparison, reliability corrections, and additional mixture and response-pattern diagnostics were introduced during peer review. The 90-item analysis was specified after the existing 83-item results were known and before the three additional fits were run. The original random halves were retained, so validation denotes an internal split rather than a newly untouched sample for all revision decisions.

### 2.2 Participants and exclusions

One of the 14,372 raw records was excluded as a hard data anomaly because it failed the scoring identity total = 90 + Σ items and contained an out-of-range value. The analytic sample comprised *N* = 14,371 complete SCL-90 records, with no missing item cells (Table 1). Location 1 included 3,540 records and Location 2 included 10,831. Because the recovered file lacked an explicit geographic codebook, these numeric groups are used for limited exploratory checks without province-specific interpretation. A retained 1/2 demographic code is left unlabeled because the source descriptions conflict about its sex or gender categories.

**Table 1:** Sample derivation and data provenance.

Step	*N*	Note
Raw records archived from Mendeley data	14,372	DOI 10.17632/btzgmr2rt2.1; CC BY 4.0
Hard anomaly excluded	−1	Failed total identity and contained an out-of-range value
Analytic sample	14,371	152 columns; 0 missing SCL-90 item cells
Location 1	3,540	Numeric grouping code; geographic label unverified
Location 2	10,831	Numeric grouping code; geographic label unverified
Calibration split	7,185	50/50 split-half design
Validation split	7,186	50/50 split-half design

The associated publication reports electronic collection during routine incoming-student assessments in October-November 2023, with different institution-level recruitment procedures (Wang et al., 2026; Methods section 2.1). The numeric location codes cannot be securely mapped to those procedures. The deposit’s undergraduate designation is retained as a source description, not an independently verified classification.

The analysis file contained no respondent identifier. Identical 90-item response patterns were therefore retained in the primary analysis: matching answers alone cannot establish duplicate participation. Their frequency, multiplicity, response characteristics, and concentration by location were assessed, and selected descriptive summaries were examined again after retaining one record per unique pattern (§3.5). This sensitivity assesses the influence of repeated patterns without redefining the primary sample or establishing duplicate participation.

### 2.3 Measure and scoring

The associated primary publication identifies its instrument as the Chinese SCL-90 translated by Wang and describes 1–5 scoring (Wang et al., 2026). The administered questionnaire and exact instructions were not recovered, so the version description is attributed to that publication rather than to an independently inspected administration form. We retain the SCL-90 designation used by the data source; studies labeled SCL-90-R are not assumed to have used identical wording. Downloaded item values were coded 0–4. We added one to each item for the primary 1–5 summaries, retaining the raw coding when specifying item-based rules.

The nine-domain mapping contains 83 items. The original scale appendix separates seven further items (19, 44, 59, 60, 64, 66, and 89) from these domains (Derogatis et al., 1973). They remain in the 90-item total and GSI and were not excluded from domain-based models because of their observed loadings or correlations. The total is the sum of all 90 recoded items (range 90–450), and the GSI is that total divided by 90 (range 1–5). The four principal structural models use the same 83 domain-scored items to compare alternative representations of the conventional domains on a common indicator set. General and specific reliability indices derived from those models refer only to that item set and specification; correlation with the 90-item total does not validate transfer of the decomposition to either 90-item score.

The retained descriptive rules were total ≥160 on the 1–5 scale, GSI ≥ 2 on the 1–5 scale, and any item ≥3 on the raw 0–4 scale, equivalent to ≥4 after recoding. The total-score cutoff has a published same-scale precedent (Zhang et al., 2025). The GSI cutoff has also been used in a Chinese student study (Li et al., 2026); this provides an operational precedent, not independent clinical or normative validation. The third rule is a study-defined high-item-response flag indicating at least one response in the upper two categories. Supplementary Table S8 retains the exact rules, existing response-pattern sensitivities, and previously computed alternative cutoffs. These proportions describe different features of the same responses. Descriptive use does not provide cross-validation, clinical cutoffs, disorder prevalence, or triage performance.

### 2.4 Analytic strategy

The sample was randomly split 50/50 with a fixed, archived seed into calibration (*n* = 7,185) and validation (*n* = 7,186) halves. This design evaluates internal stability across non-overlapping subsets of a single dataset. Analyses comprised six families.

#### 2.4.1 Reliability and descriptive flags

Cronbach’s alpha was calculated for the full 90-item scale. Alpha and one-factor maximum-likelihood omega total were calculated for each of the nine domains and the seven-item additional block; full-scale omega was not estimated. Descriptive flag proportions, overall and by location with Wilson 95% confidence intervals, are reported in Supplementary Table S8.

#### 2.4.2 Dimensional structure

Four models were fitted to the 83 domain-scored items: M1, unidimensional; M2, nine correlated factors; M3, second-order; and M4, bifactor with one general and nine orthogonal specific factors. All four were estimated with MLR in both halves and with ordinal WLSMV in validation. We reported CFI, Tucker-Lewis index (TLI), RMSEA, and standardized root mean square residual (SRMR) and distinguished convergence from targeted admissibility checks of latent matrices, extreme correlations, and item residual variances. Historical checks used the available exported matrices and parameters; the diagnostic summary distinguishes direct matrix evidence from loading-based reconstruction.

A separate analysis fitted one additional specification to all 90 items: a single general factor with uncorrelated item residuals. It was estimated sequentially with MLR in validation, MLR in calibration, and WLSMV in validation, using the original split and raw responses plus one. All three fixed the latent variance to one. MLR treated responses as numeric and excluded a mean structure; WLSMV specified all items as ordered, used delta parameterization, and included a mean structure. We retained the software’s default optimizer and tolerances, with no model modification. New fit objects were inspected for parameter and standard-error finiteness, residual and latent variances, matrix definiteness, gradients, loadings, and residual correlations. MLR CFI/TLI/RMSEA used robust fields; WLSMV used scaled fields, with 90% RMSEA intervals. The separate 90-item check evaluates strict unidimensionality; it does not estimate a 90-item general-specific reliability decomposition. Information criteria were not compared across the 83- and 90-item sets.

#### 2.4.3 Bifactor strength, reliability, and diagnostics

We calculated explained common variance (ECV), percentage of uncontaminated correlations (PUC), general omegaH, domain-specific omegaHS, factor determinacy, construct replicability (*H*), standardized general and specific loadings, item residual variances, and residual correlations (Reise, 2012; Rodriguez et al., 2016). For a domain *s*, corrected MLR omegaHS used the corresponding subscale composite variance as its denominator: (sum of specific loadings in *s*)^2^ divided by [(sum of general loadings in *s*)^2^ + (sum of specific loadings in *s*)^2^ + the sum of residual variances for items in *s*]. The submitted code had incorrectly used the variance of the full 83-item composite in this denominator. Corrected values were validated against BifactorIndicesCalculator 0.2.2. Robust delta-method 95% confidence intervals were estimated for primary MLR ECV and omega coefficients. For WLSMV, item-aligned loadings and thresholds were used for threshold-aware ordinal omega following Green and Yang (2009), reported as an estimator-sensitivity point estimate.

#### 2.4.4 Exploratory mixture-model audit

Gaussian mixtures of the nine standardized domain scores were audited for four covariance specifications (EEE, EEI, VVI, and VVV) and one to six components, yielding 24 planned model-by-component cells. To remove dependence on serialized derived-score precision, the nine mixture inputs were recomputed at runtime directly from the 83 raw item responses using the frozen domain mapping and were then standardized. The audit retained every cell, including fits that were not returned and solutions with empty hard-assignment classes. Bayesian information criterion (BIC) and sample-size-adjusted BIC were defined as lower-is-better, whereas the mclust BIC and integrated complete-data likelihood (ICL) conventions were defined as higher-is-better. We additionally fitted a fixed regularized VVV specification with multiple random starts for one to six components to assess local-maxima and start sensitivity. Substantive class interpretation was reserved for a stable, complete solution. Non-crossing hard-class means served only as descriptive summaries, leaving continuity versus subtype structure unresolved.

#### 2.4.5 Historical cross-location model sequence

The original invariance analysis used the 83-item unidimensional model (M1), MLR, and the full primary sample grouped by Location 1/2; it was not a bifactor or ordinal invariance analysis. Configural models imposed the same loading pattern, metric models constrained loadings, and scalar models additionally constrained item intercepts. The original script attempted all three levels without a stopping rule based on configural fit. We report that sequence and its adjacent changes in Supplementary Table S9 as a historical record, rather than interpreting incremental constraints without an adequate baseline (Chen, 2007; Cheung and Rensvold, 2002; Putnick and Bornstein, 2016). No new invariance models were fitted. We did not report or substantively interpret latent location-mean differences; this does not imply that no latent means were estimated under the software’s scalar-model identification.

#### 2.4.6 Sensitivity and response-pattern quality control

We examined calibration-validation agreement, MLR-WLSMV agreement, additive rescaling from 0–4 to 1–5, and selected score-level results after retaining one record per unique response pattern. The overall sensitivity retained the first occurrence of each 90-item pattern in the full sample; location-specific sensitivities independently retained the first occurrence of each pattern within each location. The existing checks recalculated full-scale alpha, the first principal-component variance of the nine domain scores, and descriptive flag rates. Pattern-deduplication sensitivity was limited to these score-level summaries; item-level CFA, bifactor, and invariance models used the primary records. Correlations of the 83-item sum with the 90-item total and of the seven-item additional sum with the core, before and after deduplication, are retained only as overlapping-score descriptions (Supplementary Table S8). They do not justify excluding items or transferring reliability estimates between item sets. Duplicate-pattern quality control recorded unique and repeated patterns, multiplicity, response characteristics, and concentration by location. The earlier 9-versus-10-indicator profile sensitivity is not interpreted substantively because the mixture audit produced no stable class solution.

### 2.5 Inference boundaries

The inferential target is the structure and model-conditional reliability of SCL-90 scores in this open dataset. Threshold exceedance and the study-defined high-item-response flag are supplementary descriptions. Disorder prevalence, diagnostic or clinical-subtype validation, predictive or service-decision thresholds, causal mechanisms, latent location-mean comparisons, and national representativeness lie outside the evidential scope.

### 2.6 Software and availability

Analyses used R 4.5.2 and lavaan 0.6-21 for CFA and invariance models (Rosseel, 2012), mclust for mixture models (Scrucca et al., 2016), base R and statistics routines for descriptives and one-factor omega, and the vendored BifactorIndicesCalculator 0.2.2 source for an independent bifactor-index check. Alpha and sensitivity calculations also used psych, and the supplementary workbook was prepared with openxlsx 4.2.8.1. Reproduction materials record package versions, analysis chronology, model specifications, diagnostics, and available warning and error logs. The submitted scripts, derived outputs, scoring rules, and split-half seed are preserved in Zenodo v1 (DOI:10.5281/zenodo.20749155; https://zenodo.org/records/20749155). Zenodo v2 (DOI:10.5281/zenodo.22148523; https://zenodo.org/records/22148523) archives the first reviewer revision’s corrections and diagnostic analyses. The additional 90-item analysis and source-qualified diagnostic summary for the present editorial revision are documented separately in the accompanying supplementary and reproduction materials; they are not contained in the existing v2 record. Respondent-level data and fitted objects containing such data are excluded from the reproduction materials. AI-assisted tools supported programming and statistical checks; their identities and uses are disclosed in the Generative AI statement.

## 3 Results

### 3.1 Sample, descriptives, and reliability

The full 90-item scale had high internal consistency (alpha = 0.978), while the nine conventional domains showed acceptable to high consistency (alpha = 0.78–0.91; omega = 0.79–0.91; Table 2). Domain means ranged from 1.31 (somatization) to 1.84 (obsessive-compulsive) on the 1–5 scale, and the GSI mean was 1.47. Supplementary Table S8 reports flag counts, proportions, Wilson intervals, and alternative-cutpoint summaries. Because these flags represent different operational definitions, their agreement cannot establish diagnostic accuracy. Likewise, high alpha for a long scale indicates internal consistency rather than unidimensionality.

**Table 2:** Reliability and descriptive statistics.

Scale	*k*	Alpha	Omega	Mean 1–5	SD 1–5
All 90 items	90	0.978	—	1.47	0.47
Somatization	12	0.874	0.884	1.31	0.43
Obsessive-compulsive	10	0.864	0.864	1.84	0.66
Interpersonal sensitivity	9	0.855	0.864	1.58	0.61
Depression	13	0.905	0.910	1.51	0.59
Anxiety	10	0.865	0.888	1.45	0.52
Hostility	6	0.804	0.825	1.34	0.49
Phobic anxiety	7	0.796	0.803	1.33	0.49
Paranoid ideation	6	0.783	0.793	1.39	0.52
Psychoticism	10	0.839	0.841	1.39	0.50
Additional items	7	0.743	0.751	1.40	0.49

Mean and SD summarize participants’ item-mean scores on the 1–5 scale; the all-90-item mean score is the GSI. Omega is one-factor maximum-likelihood omega total for each domain or additional-item block, not bifactor omegaHS. Full-scale omega was not estimated; the dash denotes not estimated.

### 3.2 Dimensional structure and the general severity factor

The nine domains were strongly intercorrelated (Pearson *r* = 0.61–0.84; highest for depression-anxiety, *r* = 0.84; Table 3), and a principal component analysis (PCA) of domain scores yielded a dominant first component (76.0% of variance, second component 4.9%, Figure 1; Table 4). The four-model comparison used the same 83 domain-scored items. Under MLR, M2 converged, but its latent correlation matrix was non-positive definite in both halves: minimum eigenvalues were −0.00990 and −0.01759, even though every individual correlation was below one. M3 passed the targeted matrix checks and served as the principal alternative to M4. M4 improved robust CFI over M3 by 0.0242 in calibration and 0.0250 in validation, while absolute fit remained mixed (validation CFI = 0.849, TLI = 0.842, RMSEA = 0.045, SRMR = 0.042; Table 5). Its structural interpretation depends on the accompanying reliability and residual evidence as well as relative fit.

**Table 3:** Dimension intercorrelations, lower triangle.

Dimension	SOM	O-C	I-S	DEP	ANX	HOS	PHOB	PAR	PSY
SOM	1.000	—	—	—	—	—	—	—	—
O-C	0.633	1.000	—	—	—	—	—	—	—
I-S	0.627	0.795	1.000	—	—	—	—	—	—
DEP	0.695	0.801	0.831	1.000	—	—	—	—	—
ANX	0.759	0.783	0.800	0.840	1.000	—	—	—	—
HOS	0.656	0.650	0.714	0.727	0.729	1.000	—	—	—
PHOB	0.617	0.633	0.726	0.687	0.714	0.613	1.000	—	—
PAR	0.643	0.701	0.796	0.771	0.765	0.744	0.644	1.000	—
PSY	0.697	0.753	0.811	0.834	0.819	0.711	0.670	0.796	1.000

SOM, somatization; O-C, obsessive-compulsive; I-S, interpersonal sensitivity; DEP, depression; ANX, anxiety; HOS, hostility; PHOB, phobic anxiety; PAR, paranoid ideation; PSY, psychoticism.

**Figure 1:**
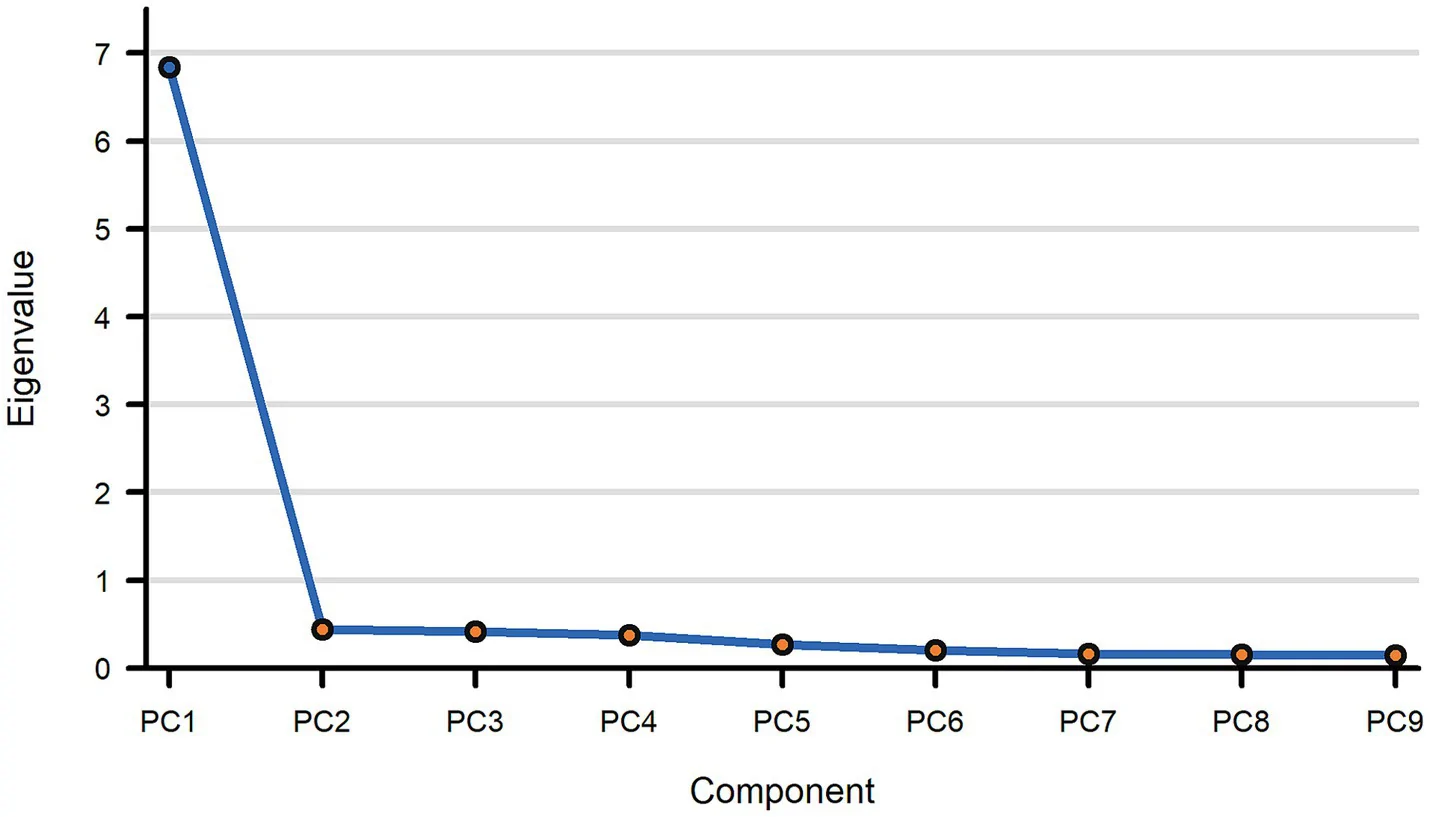
Dimension-level scree plot. The first principal component explains 76.0% of the variance and the second explains 4.9%.

**Table 4:** PCA of nine dimension scores.

Component	Eigenvalue	Variance explained	Cumulative
PC1	6.837	76.0%	76.0%
PC2	0.445	4.9%	80.9%
PC3	0.416	4.6%	85.5%
PC4	0.373	4.1%	89.7%
PC5	0.267	3.0%	92.6%
PC6	0.201	2.2%	94.9%
PC7	0.161	1.8%	96.7%
PC8	0.157	1.7%	98.4%
PC9	0.143	1.6%	100.0%

**Table 5:** MLR CFA comparison with convergence and admissibility distinguished.

Half	Model	Converged	Admissible	CFI	TLI	RMSEA [90% CI]	SRMR	BIC	Note
Calibration	M1	Yes	Yes	0.772	0.766	0.055 [0.054, 0.055]	0.049	1,144,419	None
Calibration	M2	Yes	No	0.831	0.824	0.047 [0.047, 0.048]	0.047	1,126,368	Non-PD (−0.00990)
Calibration	M3	Yes	Yes	0.821	0.816	0.048 [0.048, 0.049]	0.047	1,129,197	None
Calibration	M4	Yes	Yes	0.845	0.837	0.046 [0.045, 0.046]	0.043	1,122,121	None
Validation	M1	Yes	Yes	0.777	0.771	0.054 [0.053, 0.054]	0.048	1,130,107	None
Validation	M2	Yes	No	0.834	0.828	0.047 [0.046, 0.047]	0.045	1,112,681	Non-PD (−0.01759)
Validation	M3	Yes	Yes	0.824	0.820	0.048 [0.047, 0.048]	0.046	1,115,440	None
Validation	M4	Yes	Yes	0.849	0.842	0.045 [0.044, 0.045]	0.042	1,108,151	None

Tables 5 and 6 use the same 83 items. M1, unidimensional; M2, nine correlated factors; M3, second-order; M4, orthogonal bifactor. MLR CFI/TLI/RMSEA use robust fields; WLSMV uses scaled fields. Non-PD denotes a non-positive-definite latent correlation matrix; parenthetical values are minimum eigenvalues. Admissibility and None refer to targeted checks, not global model validity or exhaustive reinspection of historical fitted objects. M2/M3 matrix evidence comes from exported latent matrices. For ordinal M1/M4, residual checks were reconstructed from exported standardized loadings and fixed model constraints; their convergence flags alone are not admissibility evidence.

Under WLSMV, M4 had CFI = 0.925, TLI = 0.921, RMSEA = 0.040, and SRMR = 0.044, compared with M3’s 0.917, 0.915, 0.042, and 0.046 (Table 6). Its CFI advantage, calculated from the exported values, was 0.0079. M2’s CFI was only 0.0036 below M4’s, but its latent correlation matrix was non-positive definite (minimum eigenvalue −0.02065; maximum absolute off-diagonal correlation 0.991). M2 was retained as a reported comparison with an inadmissible solution. M4 also used 74 more free parameters than M3 and 47 more than M2. Interpretation of these small ordinal-fit differences also considered model flexibility, ancillary indices, residual diagnostics, and the modest MLR CFI/TLI. MLR robust and WLSMV scaled indices were evaluated separately, without cross-estimator ranking.

**Table 6:** Complete WLSMV CFA comparison in the validation half.

Model	Converged	Admissible	CFI	TLI	RMSEA	SRMR	npar	Note
M1	Yes	Yes	0.896	0.893	0.047	0.052	415	None
M2	Yes	No	0.921	0.919	0.041	0.044	451	Non-PD
M3	Yes	Yes	0.917	0.915	0.042	0.046	424	None
M4	Yes	Yes	0.925	0.921	0.040	0.044	498	None

Tables 5 and 6 use the same 83 items. M1, unidimensional; M2, nine correlated factors; M3, second-order; M4, orthogonal bifactor. MLR CFI/TLI/RMSEA use robust fields; WLSMV uses scaled fields. Non-PD denotes a non-positive-definite latent correlation matrix; parenthetical values are minimum eigenvalues. Admissibility and None refer to targeted checks, not global model validity or exhaustive reinspection of historical fitted objects. M2/M3 matrix evidence comes from exported latent matrices. For ordinal M1/M4, residual checks were reconstructed from exported standardized loadings and fixed model constraints; their convergence flags alone are not admissibility evidence.

Across calibration and validation, standardized item-loading ranges were 0.395–0.745/0.403–0.750 for M1, 0.399–0.779/0.410–0.780 for M2, and 0.403–0.776/0.412–0.780 for the first-order part of M3; M3 second-order loadings were 0.825–0.986/0.834–0.979 (Supplementary Table S7). The targeted admissibility classifications in Tables 5, 6 drew on historical convergence records, exported latent matrices, and loading-based residual checks. These classifications relied on archived exports rather than direct reinspection of the historical fitted objects. In particular, ordinal M1/M4 residual checks were reconstructed from loadings and fixed model constraints, rather than inferred from convergence alone.

Corrected bifactor indices indicated a strong general component within the 83-item specification (Table 7). Under MLR, general ECV was 0.816 [0.807, 0.825] in calibration and 0.813 [0.804, 0.822] in validation; general omegaH was 0.962 [0.960, 0.964] and 0.963 [0.961, 0.965], respectively. ECV describes the general factor’s share of modeled common item variance, whereas omegaH describes its contribution to the variance of the specified composite. Both concern variance components within the fitted model, with global fit assessed separately. Domain-specific omegaHS ranged from 0.004 to 0.306 in calibration and from 0.017 to 0.293 in validation. In validation, somatization was 0.293 [0.271, 0.315], phobic anxiety 0.251 [0.228, 0.274], and hostility 0.220 [0.194, 0.246], compared with 0.035 [0.020, 0.050] for interpersonal sensitivity and 0.017 [0.009, 0.025] for psychoticism (Supplementary Table S2). Reliable specific information varied across domains, precluding a uniform interpretation of subscale distinctiveness. WLSMV gave ECV = 0.858 and threshold-aware ordinal general omegaH = 0.967; specific omegaHS ranged from 0.005 to 0.186, with somatization (0.186), phobic anxiety (0.140), and hostility (0.111) again among the larger estimates. PUC was 0.892 throughout.

**Table 7:** Corrected bifactor strength and reliability indices.

Estimator/Half	ECV [95% CI]	omegaH [95% CI]	PUC	FD	*H*	General loading range	Specific omegaHS range	Omega method
MLR calibration	0.816 [0.807, 0.825]	0.962 [0.960, 0.964]	0.892	0.986	0.980	0.397–0.753	0.004–0.306	Standardized continuous-item omega
MLR validation	0.813 [0.804, 0.822]	0.963 [0.961, 0.965]	0.892	0.987	0.980	0.404–0.755	0.017–0.293	Standardized continuous-item omega
WLSMV validation	0.858	0.967	0.892	0.992	0.989	0.483–0.855	0.005–0.186	Threshold-aware Green-Yang ordinal omega

All rows pertain to the 83 domain-scored items and do not directly estimate reliability of the conventional 90-item total or GSI. MLR confidence intervals use the robust delta method. WLSMV omega coefficients are estimator-sensitivity point estimates without confidence intervals. ECV, explained common variance; PUC, percentage of uncontaminated correlations; FD, factor determinacy; *H*, construct replicability.

General loadings were positive in all three bifactor solutions (MLR ranges 0.397–0.753 and 0.404–0.755; WLSMV 0.483–0.855). Specific loadings varied more widely (MLR − 0.186–0.589 and −0.197–0.611; WLSMV -0.311–0.645): 10 and 11 of the 83 MLR specific loadings were negative, and 24 and 27 had absolute values below 0.10. General-factor determinacy was 0.986/0.987 under MLR and 0.992 under WLSMV; general *H* was 0.980/0.980 and 0.989. Inspected and reconstructed item residual variances were nonnegative. Local dependence remained in the residual correlations: 104 of 3,403 pairs in calibration and 86 of 3,403 in validation exceeded |0.10|, with maxima of 0.263 and 0.262; most larger residual correlations crossed conventional domains. The internally stable general-factor pattern thus coexisted with domain-specific and local structure, while external replicability remained untested (Supplementary Tables S1–S3).

The separate 90-item single-factor model converged in all three planned fits and passed the inspected numerical checks (Table 8; Supplementary Table S10). MLR calibration and validation yielded CFI = 0.769/0.773, TLI = 0.764/0.768, RMSEA = 0.052/0.052, and SRMR = 0.047/0.046. Ordinal validation yielded CFI = 0.896, TLI = 0.893, RMSEA = 0.044, and SRMR = 0.050. Positive general loadings coexisted with residual dependence: 158 and 147 of 4,005 MLR item pairs exceeded |0.10|, with maxima 0.289 and 0.319; the corresponding ordinal figures were 191 pairs and 0.315. The RMSEA and SRMR values coexisted with limited CFI/TLI and local misfit, providing insufficient support for the strict 90-item single-factor representation. More complex general-specific structures for all 90 items remained open, and a 90-item reliability decomposition was not estimated. The original warning record for the first MLR fit was incomplete; Supplementary Table S10 describes this limitation and the numerical checks of the saved models.

**Table 8:** Separate single-factor checks of the complete 90-item inventory.

Estimator/Half	*N*	Scaled Chi-square	d*f*	npar	CFI	TLI	RMSEA [90% CI]	SRMR
MLR calibration	7,185	42,478.12	3,915	180	0.769	0.764	0.052 [0.052, 0.053]	0.047
MLR validation	7,186	41,687.71	3,915	180	0.773	0.768	0.052 [0.051, 0.052]	0.046
WLSMV validation	7,186	58,959.58	3,915	450	0.896	0.893	0.044 [0.044, 0.045]	0.050

Each row fits one general factor to S1-S90, with latent variance fixed to one and all item residual covariances fixed to zero. MLR uses numeric items without a mean structure and robust CFI/TLI/RMSEA; WLSMV uses ordered items, delta parameterization, a mean structure, and scaled CFI/TLI/RMSEA. The three fits converged and passed the inspected numerical checks; this does not imply adequate absolute fit. Supplementary Table S10 reports loadings, residuals, matrix diagnostics, and the first fit’s incomplete original warning record. These results neither estimate 90-item omegaH/ECV nor warrant information-criterion comparisons with the 83-item models.

### 3.3 Exploratory mixture-model audit

The complete default enumeration contained 24 planned covariance-by-component cells. Sixteen returned a fit and only 12 met the basic admissibility checks (Supplementary Table S4). VVI and VVV fits were not returned for three to six components, and EEE solutions for three to six components contained empty hard-assignment classes. The comparison includes both missing fits and empty solutions alongside admissible results.

A fixed regularized VVV specification with multiple random starts likewise yielded no defensible stopping point. Lower-is-better BIC continued to improve from one through six components, while solutions with more components showed substantial variation in log likelihood and assignment agreement across starts. The audit therefore documents model instability without yielding a class solution for substantive interpretation. Ordered or non-crossing hard-class means under a particular fit remain descriptive summaries and leave continuity versus subtype structure unresolved.

### 3.4 Exploratory regional robustness

The 83-item M1 configural model under MLR had CFI = 0.792, RMSEA = 0.053, and SRMR = 0.044, leaving the cross-location baseline unsatisfactory. The original script had proceeded through metric and scalar models without a configural-fit stopping rule; Supplementary Table S9 therefore preserves all three fits and their adjacent differences as a historical sequence, without sequential invariance claims. This conclusion is specific to the tested unidimensional representation rather than every SCL-90 structure. Location-specific descriptive flags remain in Supplementary Table S8, without geographic or latent mean interpretation.

### 3.5 Sensitivity analyses

Among 14,371 records, 13,280 unique 90-item response patterns were observed. There were 112 distinct repeated patterns involving 1,203 records and 1,091 records in excess of one copy per pattern (Table 9; Supplementary Tables S5, S6). The largest pattern occurred 726 times and consisted of zero on all 90 raw items; it appeared 460 times in Location 1 and 266 times in Location 2. Duplicate excess was more concentrated in Location 1 (612/3,540, 17.29%) than in Location 2 (424/10,831, 3.91%). Fifty-five repeated patterns occurred in both locations and 57 in only one. The concentration of repeated patterns is a material quality-control finding, although the absence of respondent identifiers prevents it from establishing duplicated participation.

**Table 9:** Repeated response-pattern quality control.

Metric	Overall	Location 1	Location 2
Records	14,371	3,540	10,831
Unique patterns	13,280	2,928	10,407
Distinct repeated patterns	112	49	70
Records in repeated patterns	1,203	661	494
Duplicate-excess records	1,091	612	424
Duplicate excess, % of records	7.59%	17.29%	3.91%
Largest pattern multiplicity	726	460	266

Overall distinct repeated patterns have multiplicity ≥2 in the full sample, whereas location-specific counts use multiplicity ≥2 within each location; therefore, 49 + 70 need not equal 112. Fifty-five repeated patterns occurred in both locations and 57 in one location only. The largest pattern contained zero on all 90 raw items. Without respondent identifiers, repeated patterns cannot be equated with duplicated participants.

Retaining one record per unique pattern changed full-scale alpha from 0.978 to 0.976 and first-component variance from 76.0 to 74.7% (Table 10). Supplementary Table S8 reports the overall and location-specific flag sensitivities and overlapping-score correlations. These results describe selected score-level sensitivities; item-level models were not refitted after deduplication. Adding one to every raw item changes score origins and threshold labels while leaving covariance-based structure unchanged. That invariance follows from the coding operation rather than providing independent psychometric validation. Agreement between random halves likewise concerns internal stability within this dataset, with replication in a new cohort still needed.

**Table 10:** Selected score and response-pattern sensitivity.

Check	Primary analysis	One record per unique pattern	Difference or note
Full-scale alpha	0.978	0.976	−0.002
First-component variance	76.0%	74.7%	−1.3 percentage points
Item-level CFA/bifactor/invariance	Primary records only	Not refitted	Deduplication sensitivity was limited to selected score-level summaries
0–4 versus 1–5 covariance structure	Not applicable	Not applicable	Adding 1 to every item leaves covariances unchanged; threshold definitions differ

Overall deduplication retained the first occurrence of each 90-item pattern in the full sample. Flag sensitivities and overlapping-score correlations are reported in Supplementary Table S8.

## 4 Discussion

### 4.1 Principal findings

The principal finding was substantial shared general-severity variance across the 83 domain-scored items under the bifactor specification, together with limited but heterogeneous specific information. The combined loading, reliability, and residual profile supports this interpretation: the ordinal fit advantage over the second-order model was small, and MLR absolute fit remained modest. The corrected omegaHS estimates showed that somatization, phobic anxiety, and hostility retained more specific information than several other domains. The separate 90-item single-factor checks further distinguished high internal consistency and positive loadings from adequate global fit and low residual dependence. The mixture audit yielded no stable class solution, and the historical cross-location sequence lacked an adequate unidimensional configural baseline. These findings define a model-conditional account of score interpretation within this dataset.

### 4.2 Measurement implications

High general omegaH and ECV characterize complementary aspects of the fitted 83-item model (Reise et al., 2013; Rodriguez et al., 2016). Under the standardized-item MLR formulation, omegaH attributes most composite variance to the general factor, whereas ECV identifies most common item variance as general rather than specific. Positive general loadings, high determinacy, and high *H* reinforce the coherence of this representation. The narrow MLR confidence intervals describe estimation uncertainty conditional on the model and records; model misspecification and transport to other cohorts remain separate uncertainties. These estimates concern the modeled composite, not the 90-item total or GSI. The near-unity correlation with the 83-item sum reflects extensive score overlap, providing neither independent support for transferring reliability nor a basis for recommending a proxy score.

The specific-factor estimates favor a differentiated view of the nine domains: they are neither equally distinct nor uniformly uninformative. The relative prominence of somatization, phobic anxiety, and hostility resembles the pattern in Lignier et al. (2024), whose ESEM specification and item coverage nevertheless differ. Weak or negative specific loadings and cross-domain residual correlations qualify this resemblance. Domain scores describe symptom content, while substantial shared general variance and uneven omegaHS limit their interpretation as independent constructs. The bifactor account therefore concerns the distribution of modeled variance; it neither establishes a single underlying psychological entity nor uniquely selects this structure over all alternatives (Bonifay et al., 2017; Bornovalova et al., 2020; Watts et al., 2024).

The full-inventory analysis directly evaluated whether one factor with uncorrelated item residuals could adequately represent all 90 items. Under both estimators, the fit vector and residual structure provided insufficient support for that strict representation, despite positive general loadings. Substantial shared distress can thus coexist with additional specific or local covariance. The defensible form of a richer 90-item structure, and the reliable general-factor contribution to the conventional total or GSI, remain open questions. Resolving them requires a separately justified model and direct evaluation of the particular score, rather than transfer from the existing item set.

### 4.3 Descriptive screening flags and model-dependent partitions

The total-score and GSI cutpoints have published usage precedents, whereas the high-item-response flag is study-defined. Clinical and decision-criterion validity were not assessed here. The rules quantify different response features on different scales and cutpoints, so one cannot validate another. Supplementary Table S8 presents their exact definitions and results. Wilson intervals describe binomial-model precision conditional on the records and an independence assumption; population representativeness and diagnostic accuracy remain outside that interpretation.

The mixture audit showed how strongly class solutions depended on model specification. Failed high-component fits, empty hard-assignment classes, continuing BIC improvement under a fixed specification, and sensitivity to random starts all contributed to this instability. Non-crossing class means describe particular fitted partitions but cannot distinguish a continuous distribution from latent subtypes. The resulting evidence concerns model instability, with neither a retained person-centered solution nor a class-based screening recommendation.

### 4.4 Regional boundary conditions

The historical invariance sequence concerned the 83-item unidimensional model across two locations using MLR. Its inadequate configural baseline limits interpretation before metric or scalar constraints are considered (Putnick and Bornstein, 2016). Proceeding through those constraints was a limitation of the original procedure; it did not establish an acceptable baseline. A fuller assessment requires a defensible within-group representation before appropriate constraints are evaluated across documented groups. The location code permits grouping, although its geographic labels remain unconfirmed, and a second binary demographic code has conflicting source descriptions. The present result therefore applies to the tested representation, without establishing non-invariance of all SCL-90 structures or latent location-mean differences.

### 4.5 Reproducibility and open data

Versioned code and outputs document the scoring rules, model specifications, package versions, available warnings, and analyses added during peer review. They preserve unsuccessful mixture fits alongside returned solutions and distinguish model convergence from the available numerical checks. The corrected omegaHS calculation is documented with its subscale-specific denominator and an independent implementation check. These materials support examination of both the reported results and the decisions underlying this non-preregistered secondary analysis (Hardwicke et al., 2021; Nosek et al., 2015; Van den Akker et al., 2021; Wilkinson et al., 2016).

### 4.6 Limitations

The cross-sectional design and available variables constrain the uses of these findings. Without external diagnostic, prospective, or service-use criteria, the data support descriptive flagging but not clinical decisions, operational use, prevalence, or caseness estimates. The sample comes from one open dataset and is not nationally representative. Collection details and the undergraduate designation rely on the source publication; exact instructions, response rates, and voluntariness remain unverified. Location labels are unconfirmed, and conflicting descriptions prevent labeling the 1/2 demographic code as sex or gender. Without respondent identifiers, repeated patterns cannot be classified definitively as duplicate participants. Deduplication sensitivity covered selected score-level summaries rather than item-level model refitting.

Measurement invariance was examined only across locations, using the 83-item unidimensional M1 under MLR, without establishing a satisfactory configural baseline. A deeper assessment requires appropriate structures and documented groups; this result alone cannot establish non-invariance of every SCL-90 representation. Both random halves come from a single source and provide internal rather than external replication. Independent samples, different cohorts, and independently documented administration settings are needed to evaluate transportability.

Several model-dependent uncertainties remain. General and specific reliability coefficients are conditional on the 83-item bifactor specification. Modest MLR absolute fit, the small WLSMV advantage over M3, weak specific loadings, and residual local dependence constrain stronger structural claims (Bonifay et al., 2017; Bornovalova et al., 2020; Watts et al., 2024). The added 90-item model evaluated strict unidimensionality alone, leaving alternative complete-inventory structures and their score-reliability decompositions unexamined. Historical model checks relied on available exported matrices and parameters rather than new inspection of the original fit objects. Threshold-aware WLSMV omega coefficients remain sensitivity point estimates without confidence intervals.

## 5 Conclusion

In this open dataset of Chinese first-year undergraduates, the bifactor representation of the 83 domain-scored SCL-90 items combined a strong general-severity component with uneven specific information and residual dependence. The complete 90-item inventory departed from a strict single-factor representation. Together, these findings distinguish shared symptom variance from strict unidimensionality and tie reliability interpretation to the modeled score. Independent domain constructs and transfer of the 83-item decomposition to the conventional total or GSI remain unsupported. Threshold results remain non-clinical descriptions, the mixture audit supports no retained class solution, and the findings require replication in independent samples.

## Data Availability

The original dataset is available from Mendeley Data: Wang (2025), Large sample dataset of Chinese college student (SCL-90+mindfulness), V1, doi: 10.17632/btzgmr2rt2.1, under a CC BY 4.0 license. Code, scoring rules, derived tables, and reproduction materials from the first reviewer revision are archived in Zenodo v2: https://zenodo.org/records/22148523; doi: 10.5281/zenodo.22148523. The submitted v1 materials remain preserved at https://zenodo.org/records/20749155. The additional 90-item analysis and diagnostic-source update accompany the present editorial revision as separate reproduction materials; the existing v2 DOI refers to the earlier archive. The cleaned file is a derived analysis output, and the original dataset should be cited via Mendeley Data.

## References

[ref1] Bauer D. J. Curran P. J. (2003). Distributional assumptions of growth mixture models: implications for overextraction of latent trajectory classes. Psychol. Methods 8, 338–363. doi: 10.1037/1082-989X.8.3.338, 14596495

[ref2] Bonifay W. Lane S. P. Reise S. P. (2017). Three concerns with applying a bifactor model as a structure of psychopathology. Clin. Psychol. Sci. 5, 184–186. doi: 10.1177/2167702616657069

[ref3] Bornovalova M. A. Choate A. M. Fatimah H. Petersen K. J. Wiernik B. M. (2020). Appropriate use of bifactor analysis in psychopathology research: appreciating benefits and limitations. Biol. Psychiatry 88, 18–27. doi: 10.1016/j.biopsych.2020.01.013, 32199605 PMC10586518

[ref4] Chen F. F. (2007). Sensitivity of goodness of fit indexes to lack of measurement invariance. Struct. Equ. Model. Multidiscip. J. 14, 464–504. doi: 10.1080/10705510701301834

[ref5] Chen I.-H. Lin C.-Y. Zheng X. Griffiths M. D. (2020). Assessing mental health for China’s police: psychometric features of the self-rating depression scale and symptom checklist 90-revised. Int. J. Environ. Res. Public Health 17:2737. doi: 10.3390/ijerph17082737, 32316098 PMC7215956

[ref6] Cheung G. W. Rensvold R. B. (2002). Evaluating goodness-of-fit indexes for testing measurement invariance. Struct. Equ. Model. Multidiscip. J. 9, 233–255. doi: 10.1207/S15328007SEM0902_5

[ref7] Choi J. Hong S. (2022). The impact of imposing equality constraints on residual variances across classes in latent profile analysis. Front. Psychol. 12:736132. doi: 10.3389/fpsyg.2021.736132 PMC8829145 35153888

[ref8] Dang W. Xu Y. Ji J. Wang K. Zhao S. Yu B. . (2021). Study of the SCL-90 scale and changes in the Chinese norms. Front. Psychiatry 11:524395. doi: 10.3389/fpsyt.2020.524395, 33584353 PMC7873442

[ref9] Derogatis L. R. Lipman R. S. Covi L. (1973). SCL-90: an outpatient psychiatric rating scale—preliminary report. Psychopharmacol. Bull. 9, 13–28. 4682398

[ref10] Gomez R. Karimi L. Hein K. Papapetrou S. Stavropoulos V. (2026). Symptom checklist-90-revised: factor structure and relevance as a measure for the hierarchical taxonomy of psychopathology model. Int. J. Ment. Health 55, 41–64. doi: 10.1080/00207411.2024.2347188

[ref11] Green S. B. Yang Y. (2009). Reliability of summed item scores using structural equation modeling: an alternative to coefficient alpha. Psychometrika 74, 155–167. doi: 10.1007/s11336-008-9099-3

[ref12] Hardwicke T. E. Bohn M. MacDonald K. Hembacher E. Nuijten M. B. Peloquin B. N. . (2021). Analytic reproducibility in articles receiving open data badges at the journal psychological science: an observational study. R. Soc. Open Sci. 8:201494. doi: 10.1098/rsos.201494, 33614084 PMC7890505

[ref13] Li Y. Xiao G. Xue H. (2026). Heterogeneous mental health trajectories in college students: a three-year longitudinal study using latent class growth modeling. BMC Psychol. 14:883. doi: 10.1186/s40359-026-04581-8, 42063126 PMC13274059

[ref14] Lignier B. Petot J.-M. Canada B. Nicolas M. Courtois R. De Oliveira P. (2024). The structure of the symptom checklist-90-revised: global distress, somatization, hostility, and phobic anxiety scales are reliable and robust across community and clinical samples from four European countries. Psychiatry Res. 331:115635. doi: 10.1016/j.psychres.2023.115635, 38101071

[ref15] Lubke G. H. Neale M. C. (2006). Distinguishing between latent classes and continuous factors: resolution by maximum likelihood? Multivar. Behav. Res. 41, 499–532. doi: 10.1207/s15327906mbr4104_4, 26794916

[ref16] Masyn K. E. (2013). “Latent class analysis and finite mixture modeling,” in The Oxford Handbook of Quantitative Methods in Psychology, ed. Little T. D., vol. 2 (Oxford: Oxford University Press), 551–611.

[ref17] Nosek B. A. Alter G. Banks G. C. Borsboom D. Bowman S. D. Breckler S. J. . (2015). Promoting an open research culture. Science 348, 1422–1425. doi: 10.1126/science.aab2374, 26113702 PMC4550299

[ref18] Nylund K. L. Asparouhov T. Muthén B. O. (2007). Deciding on the number of classes in latent class analysis and growth mixture modeling: a Monte Carlo simulation study. Struct. Equ. Model. 14, 535–569. doi: 10.1080/10705510701575396

[ref19] Putnick D. L. Bornstein M. H. (2016). Measurement invariance conventions and reporting: the state of the art and future directions for psychological research. Dev. Rev. 41, 71–90. doi: 10.1016/j.dr.2016.06.004, 27942093 PMC5145197

[ref20] Reise S. P. (2012). The rediscovery of bifactor measurement models. Multivariate Behav. Res. 47, 667–696. doi: 10.1080/00273171.2012.715555, 24049214 PMC3773879

[ref21] Reise S. P. Bonifay W. E. Haviland M. G. (2013). Scoring and modeling psychological measures in the presence of multidimensionality. J. Pers. Assess. 95, 129–140. doi: 10.1080/00223891.2012.725437, 23030794

[ref22] Rodriguez A. Reise S. P. Haviland M. G. (2016). Evaluating bifactor models: calculating and interpreting statistical indices. Psychol. Methods 21, 137–150. doi: 10.1037/met0000045, 26523435

[ref23] Rosseel Y. (2012). Lavaan: an R package for structural equation modeling. J. Stat. Softw. 48, 1–36. doi: 10.18637/jss.v048.i02

[ref24] Scrucca L. Fop M. Murphy T. B. Raftery A. E. (2016). mclust 5: clustering, classification and density estimation using Gaussian finite mixture models. R J. 8, 289–317. doi: 10.32614/RJ-2016-021, 27818791 PMC5096736

[ref25] Urbán R. Arrindell W. A. Demetrovics Z. Unoka Z. Timman R. (2016). Cross-cultural confirmation of bi-factor models of a symptom distress measure: symptom checklist-90-revised in clinical samples. Psychiatry Res. 239, 265–274. doi: 10.1016/j.psychres.2016.03.039, 27039011

[ref26] Urbán R. Kun B. Farkas J. Paksi B. Kökönyei G. Unoka Z. . (2014). Bifactor structural model of symptom checklists: SCL-90-R and brief symptom inventory (BSI) in a non-clinical community sample. Psychiatry Res. 216, 146–154. doi: 10.1016/j.psychres.2014.01.027, 24524946

[ref27] Van den Akker O. R. Weston S. Campbell L. Chopik B. Damian R. Davis-Kean P. . (2021). Preregistration of secondary data analysis: a template and tutorial. Meta-Psychol. 5:MP.2020.2625. doi: 10.15626/MP.2020.2625

[ref28] Vassend O. Skrondal A. (1999). The problem of structural indeterminacy in multidimensional symptom report instruments. The case of SCL-90-R. Behav. Res. Ther. 37, 685–701. doi: 10.1016/S0005-7967(98)00182-X, 10402693

[ref29] Wang S. (2025). Large sample dataset of Chinese college student (SCL-90+ mindfulness). Mendeley Data, V1. doi: 10.17632/btzgmr2rt2.1

[ref30] Wang S. Chong Z. Y. Yi M. Zhang Z. Xu W. (2026). Symptom network for psychological distress in college freshmen: a large sample Bayesian network analysis. BMC Psychol. 14:737. doi: 10.1186/s40359-026-04501-w, 41965755 PMC13185341

[ref31] Watts A. L. Greene A. L. Bonifay W. Fried E. I. (2024). A critical evaluation of the p-factor literature. Nat. Rev. Psychol. 3, 108–122. doi: 10.1038/s44159-023-00260-2, 37880705

[ref32] Wilkinson M. D. Dumontier M. Aalbersberg I. J. J. Appleton G. Axton M. Baak A. . (2016). The FAIR guiding principles for scientific data management and stewardship. Sci. Data 3:160018. doi: 10.1038/sdata.2016.18, 26978244 PMC4792175

[ref33] Yu Y. Wan C. Huebner E. S. Zhao X. Zeng W. Shang L. (2019). Psychometric properties of the symptom check list 90 (SCL-90) for Chinese undergraduate students. J. Ment. Health 28, 213–219. doi: 10.1080/09638237.2018.1521939, 30465457

[ref34] Yu Y. Wan C. Zhao X. Huebner E. S. Tan J. Xu C. . (2020). Undergraduate students’ norms for the Chinese version of the symptom check-list-90-R (SCL-90-R). BMC Public Health 20:1588. doi: 10.1186/s12889-020-09689-z, 33087089 PMC7579932

[ref35] Zhang Y. He J. Ruan H. Yao P. Ma G. (2025). The impact of physical exercise on mental health and the relationship among physical exercise, emotional regulation and suicidal ideation in Chinese medical students. Front. Psychol. 16:1609415. doi: 10.3389/fpsyg.2025.1609415, 40873524 PMC12379471

[ref36] Zhou J. Yu J. Zhou Y. Qiu J. (2021). Study of item text in the Chinese symptom checklist-90. Medicine 100:e24841. doi: 10.1097/MD.0000000000024841, 33725955 PMC7982195

